# Endothelial activation and stress index for prediction of mortality in asthma

**DOI:** 10.3389/fmed.2025.1622944

**Published:** 2025-07-09

**Authors:** Yili He, Yang Li, Yuan Xiaojin, Dan Wu, Wenyan Jiang, Xianlong Xie

**Affiliations:** Department of Critical Care, Guangxi Medical University Cancer Hospital, Nanning, Guangxi, China

**Keywords:** endothelial activation and stress index, 28-day mortality, 60-day mortality, asthma, predictive performance

## Abstract

**Background:**

EASIX (Endothelial Activation and Stress Index) has been shown to be associated with the prognosis of various diseases in numerous studies, but its relationship with short- and medium-term outcomes in asthma patients admitted to the ICU (Intensive Care Unit) remains unclear.

**Methods:**

This retrospective cohort study included 3,737 asthma patients from the Medical Information Mart for Intensive Care-IV (MIMIC-IV) database (2008–2019). We calculated log2(EASIX) using platelet count, creatinine, and lactate dehydrogenase levels measured on ICU admission day 1. We analyzed the association between log2(EASIX) and 28-day and 60-day in-hospital mortality using multivariable Cox regression and restricted cubic splines. Proportional hazards assumptions were tested to ensure no time-dependent bias. Subgroup analyses and interaction tests were conducted to verify the stability. ROC (Receiver Operating Characteristic) analysis was performed to assess the prognostic performance of log2(EASIX).

**Results:**

The mean age of patients was 62.5 years, with 289 deaths at 28 days and 432 at 60 days. Higher log2(EASIX) was independently associated with increased mortality at both 28 days [hazard ratio (HR) = 1.14, 95% confidence interval (CI): 1.02–1.27, *p* = 0.017] and 60 days (HR = 1.13, 95% CI: 1.02–1.24, *p* = 0.017). The highest tertile had significantly higher mortality than the lowest tertile (28-day: HR = 1.84, 95% CI: 1.16– 2.90, *p* = 0.009; 60-day: HR = 1.65, 95% CI: 1.08–2.51, *p* = 0.019). A linear relationship was observed between log2(EASIX) and both 28-day and 60-day mortality (P for non-linearity test = 0.29 and 0.633). Subgroup analyses and interaction tests indicated that the association between log2(EASIX) and mortality was stable. The ROC curve analysis revealed AUC (Area Under the Curve) values for 28- and 60-day mortality of 0.892 and 0.881, respectively, with Youden indices of 0.63 and 0.61, indicating good predictive performance.

**Conclusion:**

Elevated log2(EASIX) levels are independently associated with increased in-hospital mortality in patients with asthma. Endothelial Activation and Stress Index show good predictive performance for short- and medium-term mortality in this patient population.

## Introduction

Asthma affects approximately 300 million people worldwide (1). It has a high prevalence and significant mortality rate. It is estimated that at least 250,000 people die from this disease worldwide each year (2). Asthma is characterized by bronchial hyperresponsiveness, reversible airflow obstruction, and chronic airway inflammation and remodeling (3). Inflammation, both local and systemic, plays a crucial role in the development and progression of asthma (4, 5). Neutrophils contribute to airway inflammation during the early stages of asthma exacerbations by releasing various pro-inflammatory cytokines and chemokines (6). Macrophages play a central role in chronic inflammation in asthma (7), releasing inflammatory molecules such as cytokines and chemokines that attract and activate other immune cells, thereby perpetuating the inflammatory response. Lymphocytes, particularly T-helper 2 (Th2) lymphocytes, release cytokines that promote airway inflammation, mucus production, and bronchoconstriction (8).

Chronic inflammatory states can lead to endothelial damage and dysfunction (9). The endothelium is a monolayer of cells lining the interior surface of blood vessels and plays a crucial role in maintaining the health of multiple organs and overall homeostasis. Healthy endothelial function includes the dynamic regulation of vascular tone, angiogenesis, hemostasis, and providing an interface with antioxidant, anti-inflammatory, and anti-thrombotic properties (10). In contrast, endothelial dysfunction is characterized by impaired endothelium-dependent vasodilation, increased oxidative stress, sustained chronic inflammation, leukocyte adhesion, and vascular hyperpermeability (11).

Endothelial cell activation and stress index are derived from hematologic patient cohorts, including lactate dehydrogenase (LDH), creatinine, and platelet levels. These biomarkers were initially observed in transplant-associated microangiopathy (TMA) and pathologic atypical hemolytic uremic syndrome (aHUS), where studies identified a consistent pattern of increased LDH and creatinine levels accompanied by decreased platelet counts. This observation led to the development of a composite biomarker known as EASIX (12), which integrates these three indicators into a continuous value. EASIX has been associated with serum NT-proBNP, soluble CD141 (sCD141, a soluble thrombomodulin) (13), angiopoietin-2 (14, 15), interleukin-18, and low levels of IGF-1 (16). These associations support the use of EASIX as a marker of endothelial cell injury. Initially, EASIX was clinically applied in hematologic conditions, such as monitoring thrombotic microangiopathy in patients undergoing allogeneic stem cell transplantation (16), predicting survival in myelodysplastic syndromes (MDS) (15), assessing endothelial injury in CAR-T cell recipients (17), and evaluating prognosis in diffuse large B-cell lymphoma (18). The application of EASIX was later expanded to assess endothelial injury in sepsis (19) and to predict outcomes in coronary artery disease with atherosclerosis. Despite ongoing debates regarding the underlying mechanisms (20), further validation of EASIX in broader populations experiencing endothelial injury remains warranted.

Currently, no studies have reported the relationship between endothelial activation and stress index and the prognosis in asthma. EASIX may be relevant to the pathogenesis of asthma. This article hypothesizes that EASIX can predict short-term outcomes in ICU patients with asthma, thus offering a potential tool for managing severe asthma cases.

## Materials and methods

### Data source

The data relevant to this study were retrospectively obtained from the Medical Information Mart for Intensive Care IV (MIMIC-IV version 2.2) database, which is accessible at https://physionet.org/content/mimiciv/2.2/. This database contains physiological information from bedside monitors in the adult intensive care unit at Beth Israel Deaconess Medical Center (BIDMC), a tertiary academic medical center in Boston, Massachusetts. The database was developed and is maintained by the MIT Laboratory for Computational Physiology and includes data from nearly 300,000 patients admitted between 2008 and 2019. We have completed the required training for accessing the database and have obtained access permission (certification number: 56396864) from Hé Yīlǐ. All protected patient information has been anonymized. This study has received ethical approval from the Medical Research Ethics Committee of Guangxi Medical University Affiliated Cancer Hospital (approval number: KY2025584). Our research report adheres to the Strengthening the Reporting of Observational Studies in Epidemiology (STROBE) guidelines.

### Patient selection

The study population included all patients from the MIMIC-IV database. Our analysis included the following patients:

1. Those diagnosed with asthma according to the International Classification of Diseases, Ninth Revision (ICD-9 codes: 493.00, 493.01, 493.02, 493.10, 493.11, 493.12, 493.20, 493.21, 493.22, 493.82, 493.90, 493.91, 493.92, 493.81) and Tenth Revision (ICD-10 codes: J45, J45.2, J45.20, J45.21, J45.22, J45.3, J45.30, J45.31, J45.32, J45.4, J45.41, J45.42, J45.5, J45.50, J45.51, J45.52, J45.9, J45.90, J45.901, J45.902, J45.909, J45.99, J45.990, J45.991, J45.998). 2. Patients aged 18 years and older. 3. Patients who stayed in the ICU for more than 24 h.

We excluded the following patients:

1. Patients with multiple admissions, retaining only the first admission record. 2. Patients whose lactate dehydrogenase, serum creatinine, and platelet levels were not measured on the first day of ICU admission. 3. Patients lacking death records.

### Data extraction

Within the first 24 h of ICU admission, we extracted baseline demographic data (age, sex, BMI, etc.), scoring systems [Charlson Comorbidity Index (CCI), Acute Physiology and Chronic Health Evaluation III (APACHE III), Sequential Organ Failure Assessment (SOFA) score], laboratory tests (complete blood count, arterial blood gas analysis, electrolytes, liver function, kidney function, coagulation profile, etc.), vital signs (heart rate, blood pressure, oxygen saturation), comorbidities (myocardial infarction, congestive heart failure, stroke, malignancy, severe liver disease, metastatic solid tumors, diabetes with complications), therapeutic interventions (antibiotic treatment, use of vasopressors, mechanical ventilation, blood purification), and outcome statistics (number of deaths at 28 and 60 days, ICU length of stay).

The endothelial activation and stress index, EASIX, was calculated using the following formula: EASIX = lactate dehydrogenase (U/L) × serum creatinine (mg/dL)/platelet count (× 10^∧^9/L).

### Missing data

The primary outcome measures—lactate dehydrogenase, serum creatinine, platelet count, number of deaths at 28 days, and number of deaths at 60 days—had no missing data. However, the missing data for some variables were as follows: albumin (ALB) had 60.11% missing, neutrophil count (NEU) had 37.20% missing, lymphocyte count (LYM) had 37.17% missing, D-dimer (D-Dimer) had 98.61% missing, prothrombin time (PT) had 13.11% missing, activated partial thromboplastin time (APTT) had 13.73% missing, lactate (LAC) had 50.84% missing, P/F ratio had 62.96% missing, total carbon dioxide (TOTAL CO_2_) had 45.60% missing, SOFA score had 54.4% missing, pH had 42.6% missing, partial pressure of carbon dioxide (PCO2) had 42.6% missing, and partial pressure of oxygen (PO2) had 42.6% missing. The remaining data had less than 1% missing or no missing values at all. Following a general data description, multiple imputation analysis was performed for the missing values, while variables with over 45% missing values were excluded from further analysis.

### Statistical analysis

We used the Kolmogorov-Smirnov test to assess the normality of continuous variables. Variables that met the criteria for normal distribution were presented as mean ± standard deviation and compared using independent samples *t*-tests. For non-normally distributed variables, data were presented as median and interquartile range (IQR) and compared using the Wilcoxon rank-sum test. Since EASIX was found to be skewed, a log transformation was applied, resulting in log2(EASIX) being normally distributed. Categorical variables were presented as counts and percentages, and comparisons were made using the chi-squared test.

We employed restricted cubic splines (RCS) to assess the correlation between log2(EASIX) and the risk of mortality at 28 and 60 days in the ICU. Additionally, Cox multivariable regression analysis was conducted to determine the association of log2(EASIX) with ICU mortality at 28 and 60 days, reporting the hazard ratios (HR) with 95% confidence intervals (CI). Log2(EASIX) levels were analyzed using tertiles. The tertile cut-off points for log_2_(EASIX) were determined empirically based on the distribution of the entire cohort. The values dividing the lowest (T1: < 0.72), middle (T2: 0.72–2.98), and highest tertiles (T3: ≥ 2.98) corresponded to the 33.3rd and 66.7th percentiles of the log_2_(EASIX) distribution, ensuring equal proportions of patients in each group. This approach is consistent with prior EASIX studies in hematologic populations and allows direct comparison of risk gradients. Both unadjusted and multivariable models were constructed. Covariates from the same analysis were stratified, and Cox regression models were used for subgroup analyses, with the results of interaction analyses presented in forest plots. Proportional hazards tests were employed to assess the time-dependent changes of covariates and the overall model.

Receiver operating characteristic (ROC) curve analysis was utilized to evaluate the predictive performance, sensitivity, and specificity of log2(EASIX) for short-term mortality in patients with asthma. The Youden index was used to determine the optimal threshold for log2(EASIX).

All analyses were conducted using the R statistical software package (version 4.4.2;R Foundation)^1^ and Free Statistics software (version 2.1), with a *p*<0.05 (two-tailed) considered statistically significant.

## Results

### Participants characteristics

**FIGURE 1:**
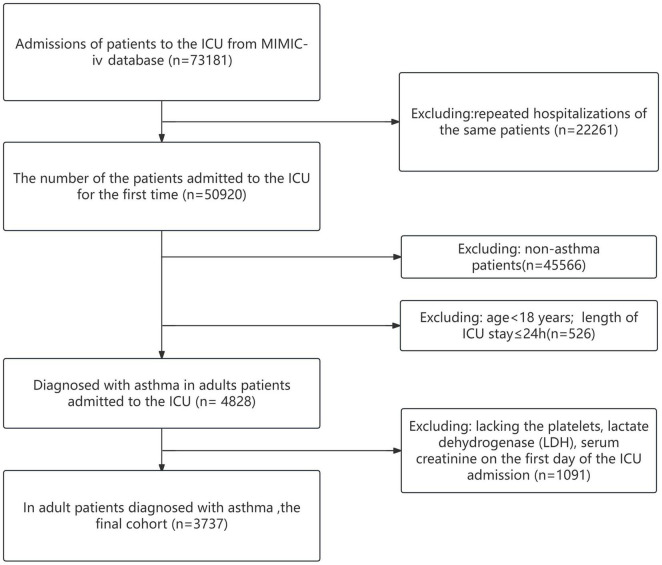
The flow chart of the study.

This study included 50,920 patients admitted to the ICU for the first time from the MIMIC-IV 2.2 database, ultimately analyzing 3,737 adult patients with asthma (see Figure 1). Among these patients, 1,517 (40.6%) were male and 2,220 (59.4%) were female, with an average age of 62.5 ± 16.0 years. The 28-day mortality rate during the ICU stay for the included patients was 7.7%, and the 60-day mortality rate was 11.6%. After stratifying patients by EASIX levels, it was observed that those with higher EASIX values had higher baseline heart rates, as well as elevated Acute Physiology Score III (APS III) and Sequential Organ Failure Assessment (SOFA) scores. The proportion of patients requiring vasopressors, mechanical ventilation, and continuous renal replacement therapy (CRRT) was also higher in this group. Additionally, levels of white blood cells, blood urea nitrogen (BUN), prothrombin time (PT), activated partial thromboplastin time (APTT), platelets, creatinine, and lactate dehydrogenase (LDH) were increased, while the oxygenation index and partial pressure of oxygen (PaO_2_) were relatively lower. The outcome events showed that both the 28-day and 60-day mortality rates increased with rising EASIX levels (see Table 1).

**TABLE 1:** Baseline characteristics of the study populations.

Variables	Total (*n* = 3737)	EASIX			*P*-value
		<1.00 (*n* = 1,246)	≥ 1.00, < 1.39 (*n* = 1,245)	≥1.39 (*n* = 1,246)	
Gender, n (%)					0.517
Female	2,220 (59.4)	729 (58.5)	735 (59)	756 (60.7)	
Male	1,517 (40.6)	517 (41.5)	510 (41)	490 (39.3)	
Age, years, Mean ± SD	62.5 ± 16.0	63.2 ± 16.1	62.6 ± 15.6	61.8 ± 16.2	0.097
BMI, kg/m^2^, Median (IQR)	30.0 (25.5, 36.1)	29.5 (25.5, 35.6)	30.2 (25.6, 36.0)	30.4 (25.4, 37.1)	0.27
**Vital sings**					
Heart_rate, Mean ± SD	86.8 ± 16.2	85.9 ± 15.7	85.9 ± 15.8	88.5 ± 17.0	<0.001
Mbp, Mean ± SD	79.9 ± 11.4	80.0 ± 11.4	79.9 ± 11.1	79.7 ± 11.9	0.781
SPO_2_, Mean ± SD	96.6 ± 2.1	96.6 ± 2.0	96.7 ± 1.9	96.6 ± 2.2	0.228
**Co-morbidities**					
Myocardial infarct, n (%)	503 (13.5)	162 (13)	151 (12.1)	190 (15.2)	0.063
Congestive heart failure, n (%)	1,095 (29.3)	331 (26.6)	346 (27.8)	418 (33.5)	<0.001
Cerebrovascular disease, n (%)	374 (10.0)	110 (8.8)	136 (10.9)	128 (10.3)	0.204
Severe liver disease, n (%)	209 (5.6)	62 (5)	65 (5.2)	82 (6.6)	0.171
Malignant cancer, n (%)	564 (15.1)	176 (14.1)	184 (14.8)	204 (16.4)	0.273
Metastatic solid tumor, n (%)	280 (7.5)	90 (7.2)	94 (7.6)	96 (7.7)	0.897
DM with complications, n (%)	498 (13.3)	170 (13.6)	167 (13.4)	161 (12.9)	0.863
**Therapies, n (%)**					
Use.hormone, n (%)	1,289 (34.5)	421 (33.8)	407 (32.7)	461 (37)	0.063
Use.vasopressin, n (%)	1,085 (29.0)	312 (25)	336 (27)	437 (35.1)	<0.001
Use.ventilation, n (%)	2,807 (75.1)	911 (73.1)	909 (73)	987 (79.2)	<0.001
Use.rrt, n (%)	223 (6.0)	59 (4.7)	61 (4.9)	103 (8.3)	<0.001
Antibiotic, n (%)	3,737 (100.0)	1,246 (100)	1,245 (100)	1,246 (100)	1
**Scores**					
Apsiii, Mean ± SD	44.9 ± 21.9	42.0 ± 20.2	43.5 ± 21.4	49.3 ± 23.5	<0.001
Sofa, Mean ± SD	3.6 ± 2.0	3.5 ± 1.9	3.5 ± 2.0	3.8 ± 2.2	0.006
**Laboratory results**					
WBC, k/μL, Median (IQR)	10.4 (7.6, 14.4)	10.2 (7.6, 13.8)	10.2 (7.6, 14.0)	11.0 (7.7, 14.9)	0.006
Neutrophils, k/μL, Median (IQR)	33.0 (8.2, 804.1)	170.2 (8.2, 776.8)	286.2 (8.4, 872.7)	17.2 (8.2, 756.5)	0.054
Lymphocytes, k/μL, Median (IQR)	5.4 (1.2, 122.6)	24.3 (1.4, 126.5)	36.4 (1.4, 136.5)	2.7 (1.0, 108.0)	<0.001
Hemoglobin, g/dL, Mean ± SD	10.7 ± 2.1	10.7 ± 2.0	10.7 ± 2.0	10.7 ± 2.2	0.598
Albumin, g/dL, Mean ± SD	3.3 ± 0.7	3.4 ± 0.6	3.4 ± 0.7	3.3 ± 0.7	0.011
Sodium, mmol/L, Mean ± SD	137.8 ± 4.5	137.7 ± 4.4	137.9 ± 4.3	137.8 ± 4.8	0.744
Chloride, mmol/L, Mean ± SD	102.9 ± 5.9	102.9 ± 5.7	103.1 ± 5.7	102.6 ± 6.2	0.061
BUN, mmol/l, Median (IQR)	18.0 (12.5, 29.0)	17.2 (12.0, 26.5)	17.5 (12.5, 29.0)	19.5 (13.1, 32.0)	<0.001
PT, sec, Mean ± SD	15.5 ± 7.4	15.0 ± 7.5	15.4 ± 7.3	16.1 ± 7.5	0.003
APTT, sec, Mean ± SD	36.7 ± 18.2	35.1 ± 16.0	36.2 ± 17.2	38.9 ± 20.7	<0.001
Lactate, mmol/L, Median (IQR)	1.9 (1.3, 2.7)	1.8 (1.3, 2.6)	1.8 (1.2, 2.6)	1.9 (1.3, 2.8)	0.113
PAO_2_/FiO_2_, Mean ± SD	266.3 ± 127.0	270.6 ± 126.9	275.0 ± 123.9	255.3 ± 129.2	0.04
PO_2_, mmHg, Mean ± SD	156.6 ± 86.9	160.5 ± 90.7	160.7 ± 87.4	149.5 ± 82.6	0.018
**Laboratory results**					
PCO_2_, mmHg, Mean ± SD	43.7 ± 11.9	43.7 ± 12.0	43.6 ± 11.9	43.9 ± 11.8	0.871
Platelets, k/μL, Mean ± SD	215.6 ± 100.8	159.7 ± 45.8	184.8 ± 64.9	302.3 ± 112.1	<0.001
Scr, mg/dL, Median (IQR)	0.9 (0.7, 1.4)	0.7 (0.7, 0.8)	1.0 (0.8, 1.1)	1.9 (1.4, 3.0)	<0.001
LDH, μ/L, Mean ± SD	266.3 ± 147.5	184.8 ± 25.3	217.4 ± 44.4	396.7 ± 191.3	<0.001
Log2.EASIX, Median (IQR)	1.1 (0.9, 1.7)	0.9 (0.8, 0.9)	1.1 (1.1, 1.2)	2.2 (1.7, 4.0)	<0.001
**Outcomes**					
mortality_28d, n (%)					<0.001
No	3,448 (92.3)	1,175 (94.3)	1,168 (93.8)	1,105 (88.7)	
Yes	289 (7.7)	71 (5.7)	77 (6.2)	141 (11.3)	
mortality_60d, n (%)					<0.001
No	3,305 (88.4)	1,138 (91.3)	1,120 (90)	1,047 (84)	
Yes	432 (11.6)	108 (8.7)	125 (10)	199 (16)	

Data Presentation: Normally distributed variables are represented as mean ± standard deviation (SD), skewed variables as median (IQR), and categorical variables as numbers (proportions). BMI, Body Mass Index; MBP, Mean Blood Pressure; SPO_2_, Peripheral Oxygen Saturation; DM, Diabetes Mellitus; RRT, Renal Replacement Therapy; APSIII, Acute Physiology Score III; SOFA, Sequential Organ Failure Assessment; WBC, White Blood Cell;BUN, Blood Urea Nitrogen; PT, Prothrombin Time; APTT, Activated Partial Thromboplastin Time; Scr, Serum Creatinine; LDH, Lactate Dehydrogenase; PaO_2_/FiO_2_, Oxygenation Index; PO_2_, Partial Pressure of Oxygen; PCO_2_, Partial Pressure of Carbon Dioxide; EASIX, Endothelial Activation and Stress Index.

### The relationship between log_2_(EASIX) and mortality rates

We established a total of four models. First, we transformed the skewed EASIX data using a logarithmic transformation to obtain a normally distributed log_2_(EASIX). We then included log_2_(EASIX) both as a continuous variable and as a categorical variable in a Cox proportional hazards regression analysis. In the analysis where log_2_(EASIX) was treated as a continuous variable, the results for 28-day mortality from the univariate analysis and Models 1 to 4 were as follows: (HR = 1.19, 95% CI: 1.08–1.31, *P* = 0.001), (HR = 1.21, 95% CI: 1.09–1.33, *P* < 0.001), (HR = 1.21, 95% CI: 1.10–1.34, *P* < 0.001), (HR = 1.17, 95% CI: 1.06–1.30, *P* = 0.003), and (HR = 1.14, 95% CI: 1.02–1.27, *P* = 0.017). The trend of the HR values was stable, indicating that for each 1-unit increase in log_2_(EASIX), the risk of mortality increased by 14% in the fully adjusted model. When log_2_(EASIX) was grouped and included in the model analysis, the differences in mortality rates between the highest and lowest groups were even more pronounced, yielding results of (HR = 1.96, 95% CI: 1.28–3.01, *P* = 0.002), (HR = 2.33, 95% CI: 1.51–3.60, *P* < 0.001), (HR = 2.29, 95% CI: 1.48–3.54, *P* < 0.001), (HR = 1.99, 95% CI: 1.28–3.10, *P* = 0.002), and (HR = 1.84, 95% CI: 1.16–2.90, *P* = 0.009). This trend was consistent with the ungrouped HR values.

In the analysis of the relationship between log_2_(EASIX) and 60-day mortality, the results from the univariate analysis and Models 1–4 were as follows: (HR = 1.15, 95% CI: 1.05–1.26, P = 0.002), (HR = 1.17, 95% CI: 1.07–1.28, P = 0.001), (HR = 1.18, 95% CI: 1.08–1.29, *P* < 0.001), (HR = 1.15, 95% CI: 1.05–1.27, *P* = 0.003), and (HR = 1.13, 95% CI: 1.02–1.24, *P* = 0.017). When log_2_(EASIX) was grouped for the model analysis, the differences in mortality rates between the highest and lowest groups were similarly significant, with results of (HR = 1.68, 95% CI: 1.13–2.49, *P* = 0.01), (HR = 2.01, 95% CI: 1.35–2.96, *P* = 0.001), (HR = 1.96, 95% CI: 1.32–2.93, *P* = 0.001), (HR = 1.79, 95% CI: 1.20–2.68, *P* = 0.004), and (HR = 1.65, 95% CI: 1.08–2.51, *P* = 0.019). The trend of the HR values was consistent with the previous models (see Table 2). These findings indicate that, after adjusting for various confounding factors, log_2_(EASIX) remains positively correlated with mortality in patients with asthma.

**TABLE 2:** Risk of 28- and 60-day Mortality according to Log2-EASIX.

Variable	Non-adjusted		Model 1		Model 2		Model 3		Model 4	
	HR (95%CI)	*P*-value	HR (95%CI)	*P-*value	HR (95%CI)	*P-*value	HR (95%CI)	*P-*value	HR (95%CI)	*P*-value
**28-day mortality**										
log2.EASIX	1.19 (1.08∼1.31)	0.001	1.21 (1.09∼1.33)	< 0.001	1.21 (1.1∼1.34)	< 0.001	1.17 (1.06∼1.3)	0.003	1.14 (1.02∼1.27)	0.017
**Tertile of log2.EASIX**										
<0.72	Reference		Reference		Reference		Reference		Reference	
0.72–2.98	1.31 (1.02∼1.7)	0.038	1.3 (1∼1.68)	0.049	1.31 (1.01∼1.7)	0.043	1.19 (0.92∼1.55)	0.19	1.08 (0.82∼1.41)	0.597
≥ 2.98	1.96 (1.28∼3.01)	0.002	2.33 (1.51∼3.6)	< 0.001	2.29 (1.48∼3.54)	< 0.001	1.99 (1.28∼3.1)	0.002	1.84 (1.16∼2.9)	0.009
**60-day mortality**										
log2.EASIX	1.15 (1.05∼1.26)	0.002	1.17 (1.07∼1.28)	0.001	1.18 (1.08∼1.29)	< 0.001	1.15 (1.05∼1.27)	0.003	1.13 (1.02∼1.24)	0.017
**Tertile of log2.EASIX**										
<0.72	Reference		Reference		Reference		Reference		Reference	
0.72–2.98	1.23 (0.98∼1.54)	0.076	1.23 (0.98∼1.55)	0.079	1.25 (0.99∼1.58)	0.055	1.17 (0.93∼1.48)	0.183	1.09 (0.86∼1.38)	0.481
≥2.98	1.68 (1.13∼2.49)	0.01	2.01 (1.35∼2.99)	0.001	1.96 (1.32∼2.93)	0.001	1.79 (1.2∼2.68)	0.004	1.65 (1.08∼2.51)	0.019

Model 1 adjusted for age, gender, BMI, heart rate, MBP and SPO_2_. Model 2 adjusted for model 1 plus mycardial infarct, congestive heart failure, cerebrovascular disease, DM with complications, severe liver disease, malignant cancer and metastatic solid tumor. Model 3 adjusted for model 2 plus Mechanical ventilation, Renal replacement therapy, use hormone, use vasopressin, and apsiii score. Model 4 adjusted for model 3 plus WBC, neutrophils, lymphocytes, hemoglobin, BUN, PT, APTT, sodium, chloride, PCO_2_, and PO_2_. BMI, Body Mass Index; MBP, Mean Blood Pressure; SPO_2_, Peripheral Oxygen Saturation; DM, Diabetes Mellitus; RRT, Renal Replacement Therapy; APSIII, Acute Physiology Score III; WBC, White Blood Cell; BUN, Blood Urea Nitrogen; PT, Prothrombin Time; APTT, Activated Partial Thromboplastin Time; EASIX, Endothelial Activation and Stress Index.

The restricted cubic splines (RCS) analysis considered various adjustment factors, including general baseline characteristics, comorbidities, severity scoring systems, major treatments upon ICU admission, and important laboratory tests. In the 28-day mortality model, the *p*-value for overall significance was 0.016, while the *p*-value for non-linearity was 0.29. In the 60-day mortality model, the *p*-value for overall significance was 0.024, and the *p*-value for non-linearity was 0.633. Based on these results, we established a linear dose-response relationship between log_2_(EASIX) and mid-term mortality in patients with asthma (see Supplementary Figures 1, 2).

The results of the proportional hazards (PH) test model, which included all covariates, are presented in Supplementary Table 1. The log_2_(EASIX) variable satisfied the PH assumption, with a *p*-value of 0.593 (*p* > 0.05). The Schoenfeld residual plots (see Supplementary Figure 3) further corroborated this finding. For the global test, the chi-square value was 39.353, with 25 degrees of freedom, and the p-value was 0.207 (*p* > 0.05) (see Supplementary Table 1).

### Subgroup analyses

**FIGURE 2:**
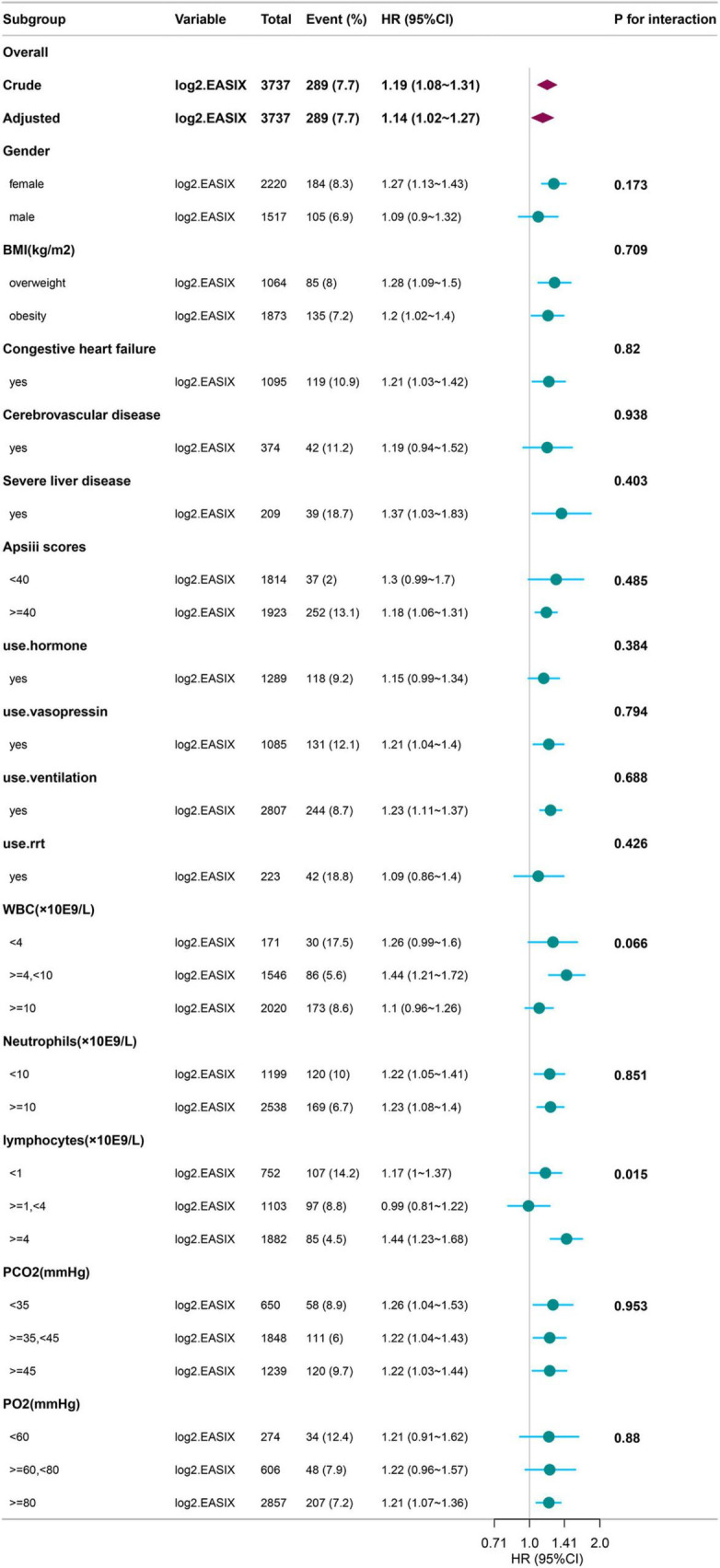
Subgroup Analysis of log2(EASIX) and 28-Day Mortality in Diseases with Asthma. BMI, Body Mass Index; RRT, Renal Replacement Therapy; APSIII, Acute Physiology Score III; WBC, White Blood Cell; PO_2_, Partial Pressure of Oxygen; PCO_2_, Partial Pressure of Carbon Dioxide; EASIX, Endothelial Activation and Stress Index.

The results of the subgroup analysis are shown in Figure 2. We conducted stratified analyses based on factors such as sex, BMI, comorbidities, APS III score, treatment methods after ICU admission, WBC, NEU, LYM, PCO_2_, and PO_2_. Significant interactions were observed only in the lymphocyte (LYM) subgroup (*p* = 0.015), while no significant interactions were noted in any other subgroup (all remaining *p* > 0.05).

### ROC curve analysis

**FIGURE 3:**
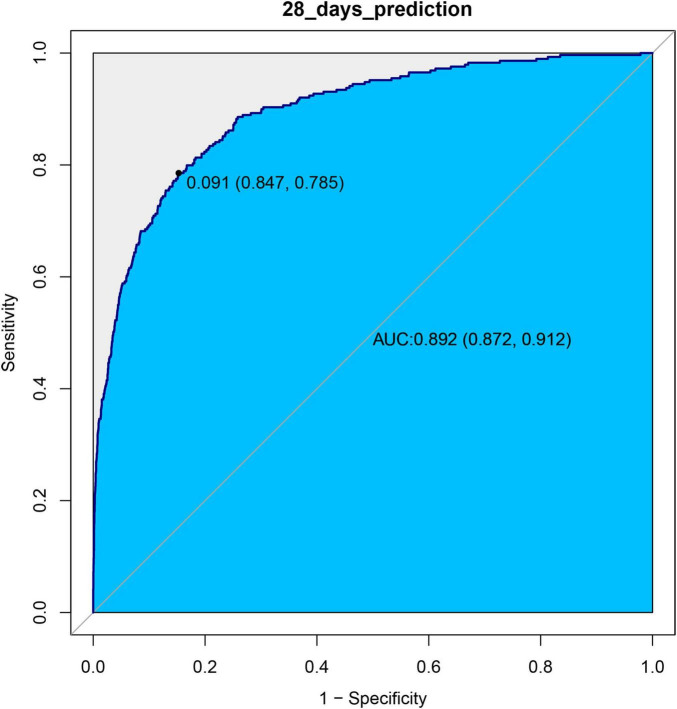
ROC curves of log2(EASIX) and 28-days mortality.

**FIGURE 4:**
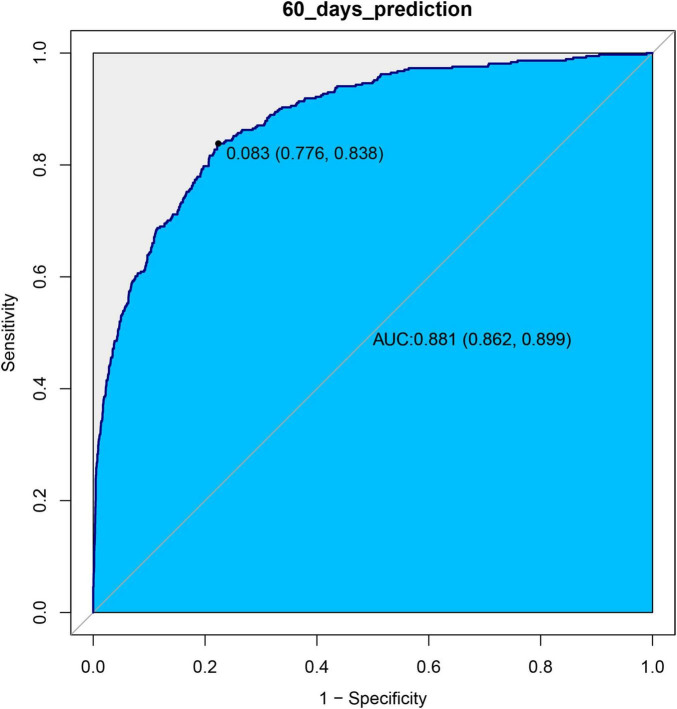
ROC curves of log2(EASIX) and 60-days mortality.

We used ROC curves to evaluate the predictive value of log_2_(EASIX) for 28-day and 60-day mortality in patients with asthma within this cohort. The AUC for log_2_(EASIX) was 0.8923 for 28-day mortality and 0.8807 for 60-day mortality (see Figures 3, 4). At the optimal cutoff values, the Youden indices for these two endpoints were 0.6326 and 0.6146, respectively (see Table 3).

**TABLE 3:** Information of receiver operating characteristic curve in Figures 3, 4.

Variable	AUC	95%CI	Threshold	Sensitivity	Specificity	Youden
Prediction for 28 days	89.23%	87.21-91.24%	0.0913	0.7855	0.8472	0.6326
Prediction for 60 days	88.07%	86.23-89.92%	0.0826	0.8383	0.7763	0.6146

## Discussion

In this retrospective cohort study, we found that EASIX levels were positively correlated with the 28-day and 60-day mortality risks among asthma patients. After considering numerous confounding factors and different time models, the association remains stable and consistent. Furthermore, forest plots demonstrated that EASIX levels had minimal interaction with potential covariates that could affect the results. As EASIX levels increased, the mortality rate rose accordiingly. When we stratified EASIX levels into tertiles, the highest level group had a higher mortality rate than the lowest level group. In the proportional hazards tests that included all examined variables, EASIX met the proportional hazards (PH) assumption, with the hazard ratio remaining constant within 28 days. Thus, it did not need to be treated as a time-dependent variable (Supplementary Table 1; Supplementary Figure 3). The global test suggested that the overall model’s proportional hazards assumption was acceptable, allowing for reliable analysis using the Cox proportional hazards model (Supplementary Table 1). Finally, we evaluated the predictive performance of log2(EASIX); both the 28-day and 60-day models showed good predictive performance (Table 3 and Figures 3, 4).

Bronchial asthma is a heterogeneous disease characterized by chronic airway inflammation and airway hyperreactivity. Its pathological features include variable and reversible airflow limitation, with later stages of the disease often leading to structural changes in the airways, known as airway remodeling (21). Currently, the diagnosis of asthma can be confirmed through a combination of pulmonary function tests and clinical symptoms. The prevalence of asthma is rising annually, with higher rates observed in developed countries compared to developing ones, and in urban areas compared to rural ones. The mortality rate ranges from 1.6 to 36.7 per 100,000, often associated with poor long-term asthma control and delays in treatment during exacerbations (22), highlighting the need for increased awareness. Recently, several new comprehensive indices have emerged to assess the prognosis of asthma. For instance, the systemic immune-inflammation index and the neutrophil-to-lymphocyte ratio have been proposed. The former is derived from the same system sample report (lymphocytes, neutrophils, and platelets) and aims to reflect the overall condition of the body. However, it acknowledges its limitations, including the lack of comparative information with other inflammatory markers (such as interleukin-6, procalcitonin, etc.), which hampers its ability to accurately reflect the inflammation status (3). The latter index also notes that it only reflects the body’s status based on the severity of inflammation and does not compare with other inflammatory parameters (like CRP) simultaneously, relying on results from a single specimen (23). In discussing the characteristics of the current study, EASIX integrates levels of lactate dehydrogenase, serum creatinine, and platelet counts. Lactate dehydrogenase is an enzyme found in many cells (such as those in the heart, skeletal muscle, liver, endothelial cells, and red blood cells) and is released upon cell damage, reflecting endothelial injury and tissue hypoxia. Serum creatinine indicates kidney function impairment (due to microvascular disease or insufficient renal perfusion), while platelets reflect coagulation status and endothelial homeostasis. This composite index provides a more comprehensive assessment of systemic endothelial injury and stress. Currently, there has been no analysis relating EASIX to asthma prognosis. The current state of research on EASIX has already been outlined in the introduction.

In the experimental data of this study, the tertile data of log2(EASIX) appear to initially demonstrate a threshold effect (Table 2). Specifically, the middle tertile group (0.72–2.98) showed no significance in Model 4 (28-day *P* = 0.597; 60-day *P* = 0.481), while the highest tertile group (≥ 2.98) exhibited a significantly increased risk, with a hazard ratio (HR) of 1.84 (95% CI: 1.16–2.90, *P* = 0.009) in the 28-day study group, and HR = 1.65 (95% CI: 1.10–2.48, *P* = 0.019) in the 60-day study group. This prompted us to further employ restricted cubic splines (RCS) to examine the non-linear relationship between log2(EASIX) and mortality. The analysis revealed a significant association between log2(EASIX) and mortality in both the 28-day and 60-day study groups, with *P*-values for the non-linear tests exceeding 0.05 (Supplementary Figures 1, 2), leading us to interpret the relationship between log2(EASIX) and both short-term and mid-term mortality as linear. The stratification of Log2-EASIX based on this relationship also follows the practices outlined in relevant literature (19). Finally, to verify the stability of the model, we performed subgroup analyses to test for interactions with the covariates. The forest plot revealed that only lymphocyte stratification showed a significant interaction (P for interaction = 0.015, Figure 2), while the remaining results remained stable. We will discuss the association mechanisms of individual variables subsequently, and the subgroup analyses suggested the robustness of the log2(EASIX) variable within the overall study. Lastly, we evaluated the predictive performance of log2(EASIX) across different time windows in both the 28- and 60-day study groups, with AUC values exceeding 0.85, indicating excellent discrimination ability. The thresholds for the two groups were 0.091 and 0.083, respectively. At these points, the corresponding sensitivity and false-positive rate (1—specificity) can be used to calculate the Youden index, which is maximized at these values, confirming its independent predictive value in short- and medium-term prognosis. Regarding the ROC curve analysis, the AUC for the 28-day model is slightly higher (0.892 vs. 0.881), which may relate to the fact that short-term mortality mechanisms are more dependent on acute pathophysiological changes, such as endothelial injury.

Although EASIX was initially validated in hematologic conditions characterized by thrombotic microangiopathy [e.g., thrombotic microangiopathy after stem cell transplantation (16)], its elevation in asthma likely reflects distinct but overlapping pathways. First, chronic airway inflammation drives endothelial dysfunction through multiple mechanisms: various inflammatory mediators (such as TNF-α, IL-1β, IL-6) can damage endothelial cells; the activation of Toll-like receptors (TLRs) and the NLRP3 inflammasome leads to endothelial dysfunction; and the NF-κB signaling pathway activates endothelial cells, upregulating adhesion molecules (such as VCAM-1, ICAM-1) (9). It is now clear that in asthma patients, the IL-33-ST2 signaling pathway in endothelial cells activates type 2 inflammatory responses, exerting pro-inflammatory effects and causing damage (24). Second, during asthma exacerbations, airway obstruction leads to hypoxia, which in turn releases hypoxia-inducible factor (HIF-1α), resulting in endothelial dysfunction (11).

Lactate is one of the end products of glycolysis, and LDH is the key enzyme that catalyzes the conversion of pyruvate to lactate. Elevated levels of LDH are typically associated with increased lactate levels and are often indicative of cellular damage and necrosis, which can lead to more severe airway inflammation in asthma patients (25). Platelets, on the other hand, may contribute to the pathogenesis of asthma by participating in airway inflammatory responses and modulating immune reactions (26). Therefore, a possible conclusion from the comparison is that in hematologic diseases, EASIX reflects thrombotic microangiopathy (e.g., complement activation), while in asthma, it is more inclined to reflect chronic inflammatory endothelial leakage.

Regarding the coupling of lactate dehydrogenase (LDH), serum creatinine, and platelet counts in EASIX, the following explanations can be considered: LDH serves as a marker for tissue cell damage, and its presence in endothelial cells means that an increase in its release reflects the disruption of the endothelial barrier. This disruption leads to the exposure of the vascular endothelial basement membrane, resulting in platelet activation and aggregation, which further triggers the release of inflammatory factors and the formation of microthrombi. These microthrombi can obstruct the renal tubules, causing microcirculatory disturbances in the kidneys, ultimately leading to an increase in serum creatinine levels. As for the potential interaction mechanism between log2(EASIX) and lymphocyte levels observed in the forest plot, we propose the following interpretations: 1. In the normal lymphocyte level group (1–4 × 10^9^/L), the body is in a state of balance between anti-inflammatory and pro-inflammatory responses. A sufficient number of lymphocytes may help maintain immune balance, counteracting the endothelial damage associated with elevated EASIX levels. Possible “threshold effect”: Within this range, lymphocytes may mitigate the negative impact of elevated EASIX levels by maintaining immune surveillance functions, such as the clearance of apoptotic endothelial cells. Consequently, the hazard ratio (HR) for log2(EASIX) is 0.99 (95% CI: 0.81–1.22), which is close to 1 and not statistically significant, indicating no notable effect on outcomes. 2. In the low lymphocyte group (< 1 × 10^9^/L), the HR for Log2-EASIX is 1.17 (95% CI: 1.00–1.37). Here, endothelial dysfunction is represented by elevated EASIX levels, and the reduction in lymphocytes suggests impaired immune function (27). This impairment is associated with endothelial system damage (28); Possible imbalance of the “immune-endothelial axis”: A reduction in lymphocytes not only reflects immune suppression but may also directly lead to a decline in endothelial repair capacity, such as reduced secretion of vascular endothelial growth factor (VEGF) or impairment of repair-related cytokines (29). These changes may contribute to increased mortality. 3. In the high lymphocyte group (≥ 4 × 10^9^/L), the elevated reactive lymphocytes are less commonly reported as being associated with heightened inflammatory responses. Some reactive increases may represent an immune response to viral infections (30), while an increase in tumor-associated lymphocytes may reflect abnormal immune suppression (31). Additionally, lymphocyte increases associated with excessive immune responses can also be observed in certain autoimmune diseases (32). Either excessively high or low immune responses may lead to more severe endothelial leakage and thrombosis (33, 34), which would be associated with high EASIX levels.

Our research reveals that the association between EASIX and medium- to short-term mortality may be modulated by lymphocyte counts, which has three key clinical implications: 1. Risk Warning: Patients with abnormal lymphocyte counts (< 1 or > 4 × 10^9^/L) should be vigilant about the increased mortality risk associated with elevated EASIX levels. It is recommended that this ipopulation undergo concurrent testing of EASIX and lymphocyte counts upon admission. The combination of EASIX and lymphocyte counts may optimize mortality risk prediction for asthma patients, particularly those with severe disease or concurrent infections or autoimmune conditions. 2. Pathological Subtyping: Lymphocyte counts > 4 × 10^9^/L may indicate a specific subtype of asthma (e.g., associated with autoimmune conditions or infections), necessitating targeted investigations to identify the underlying causes. 3. Therapeutic Targets: Future research could explore whether medications that modulate lymphocyte-endothelial interactions (such as JAK inhibitors) can improve patient outcomes.

This study also has several limitations. Although the multivariable covariates cover a wide range of influencing factors, there may still be unmeasured confounding factors (such as genetic background and specific medication dosages) that could affect the accuracy of the association between EASIX and short-term mortality in asthma patients. While the restricted cubic splines (RCS) analysis indicates a linear trend, the tertile analysis shows that only the high tertile group has a significant risk, which may suggest the presence of a clinical threshold (e.g., a sharp increase in risk when ≥ 2.98). The low incidence of endpoint events in the study population suggests that future research should expand the sample size to validate clinical applicability. The highest tertile group (≥ 2.98) could be considered for future studies to verify this threshold. While tertiles provided clinically interpretable risk stratification, future studies could explore data-driven optimal cut-offs using machine learning approaches in larger cohorts. Additionally, collecting and analyzing long-term mortality data at 90 days could increase the number of endpoint events and potentially demonstrate better clinical utility.

## Conclusion

Elevated EASIX levels are significantly associated with the risk of all-cause mortality in asthma patients at both 28 days and 60 days. EASIX demonstrates important clinical value for early risk stratification and prognostic prediction in asthma patients.

## Data Availability

The raw data supporting the conclusions of this article will be made available by the authors, without undue reservation.

## Footnotes

1
http://www.R-project.org

## Supplementary material

Supplementary Figure 1 Restricted cubic spline plot (Log2(EASIX) and 28-day mortality rate model). The variables that support the model include: Gender, age, BMI, heart rate, MBP, SPO_2_, charlson comorbidity index, apsiii, use hormone, use vasopressin, use ventlation, use rrt, wbc, neutrophils, lymphocytes, hemoglobin, BUN, PT, APTT, sodium, chloride, PCO_2_ and PO_2_.

Supplementary Figure 2 Restricted cubic spline plot (Log2(EASIX) and 60-day mortality rate model). The variables that support the model include: gender, age, BMI, heart rate, MBP, SPO_2_, charlson comorbidity index, apsiii, use hormone, use vasopressin, use ventlation, use RRT, WBC, neutrophils, lymphocytes, hemoglobin, BUN, PT, APTT, sodium, chloride, PCO_2_ and PO_2_.

Supplementary Figure 3 Schoenfeld residual plot for Log2(EASIX) in relation to 28-day mortality. A 1-unit increment in log2(EASIX) corresponded to a time-independent 14% increase in 28-day mortality risk, supporting the proportionality of hazards.
