# Supplementary material for: Endothelial activation and stress index for prediction of mortality in asthma
Source: Front Med (Lausanne). 2025 Jul 9;12:1622944. doi: 10.3389/fmed.2025.1622944 (PMC12283289; doi:10.3389/fmed.2025.1622944)
Supplement: Supplementary file 1 [file Table_1.docx]

**Supplementary table 1: The proportional hazards model variable test for the relationship between Log2-EASIX and 28-day mortality rate model**

| **Variable** | **chisq** | **df** | **p.value** |
| --- | --- | --- | --- |
| log2.EASIX | 0.286 | 1 | 0.593 |
| gender | 0.154 | 1 | 0.694 |
| Admission age | 0.002 | 1 | 0.969 |
| Heart rate mean | 2.388 | 1 | 0.122 |
| Mbp mean | 0.344 | 1 | 0.557 |
| spo2 mean | 1.216 | 1 | 0.27 |
| myocardial_infarct | 0.354 | 1 | 0.552 |
| congestive_heart_failure | 0.546 | 1 | 0.46 |
| cerebrovascular_disease | 1.305 | 1 | 0.253 |
| diabetes_with_cc | 0.44 | 1 | 0.507 |
| severe_liver_disease | 0.249 | 1 | 0.617 |
| malignant_cancer | 1.489 | 1 | 0.222 |
| metastatic_solid_tumor | 1.237 | 1 | 0.266 |
| whether.use.hormone | 2.69 | 1 | 0.101 |
| whether.use.vasopressin | 10.036 | 1 | 0.002 |
| whether.use.ventilation | 1.636 | 1 | 0.201 |
| whether.use.rrt | 0.914 | 1 | 0.339 |
| wbc_mean | 0.575 | 1 | 0.448 |
| hemoglobin_mean | 0.984 | 1 | 0.321 |
| bun_mean | 1.647 | 1 | 0.199 |
| pt_mean | 0.17 | 1 | 0.68 |
| ptt_mean | 0.653 | 1 | 0.419 |
| pco2_mean | 0.633 | 1 | 0.426 |
| po2_mean | 0.459 | 1 | 0.498 |
| BMI2530 | 2.516 | 2 | 0.284 |
| neutrophils10 | 0.133 | 1 | 0.715 |
| lymphocytes3 | 0.743 | 1 | 0.389 |
| sodium135145 | 4.791 | 2 | 0.091 |
| cholride96106 | 4.145 | 2 | 0.126 |
| apsiii40 | 1.708 | 1 | 0.191 |
| GLOBAL | 39.353 | 33 | 0.207 |

**Note**:Log2-EASIX and other variables all meet the proportional hazards (PH) assumption, except for vasopressin. This suggests that the main variables in the model have a stable impact on mortality risk within 28 days (the hazard ratio does not change over time). The global test indicates that the overall model meets the PH assumption, showing that the model is time-independent and robust for use.
