# Supplementary figures and images for: Endothelial activation and stress index for prediction of mortality in asthma
Source: Front Med (Lausanne). 2025 Jul 9;12:1622944. doi: 10.3389/fmed.2025.1622944 (PMC12283289; doi:10.3389/fmed.2025.1622944)

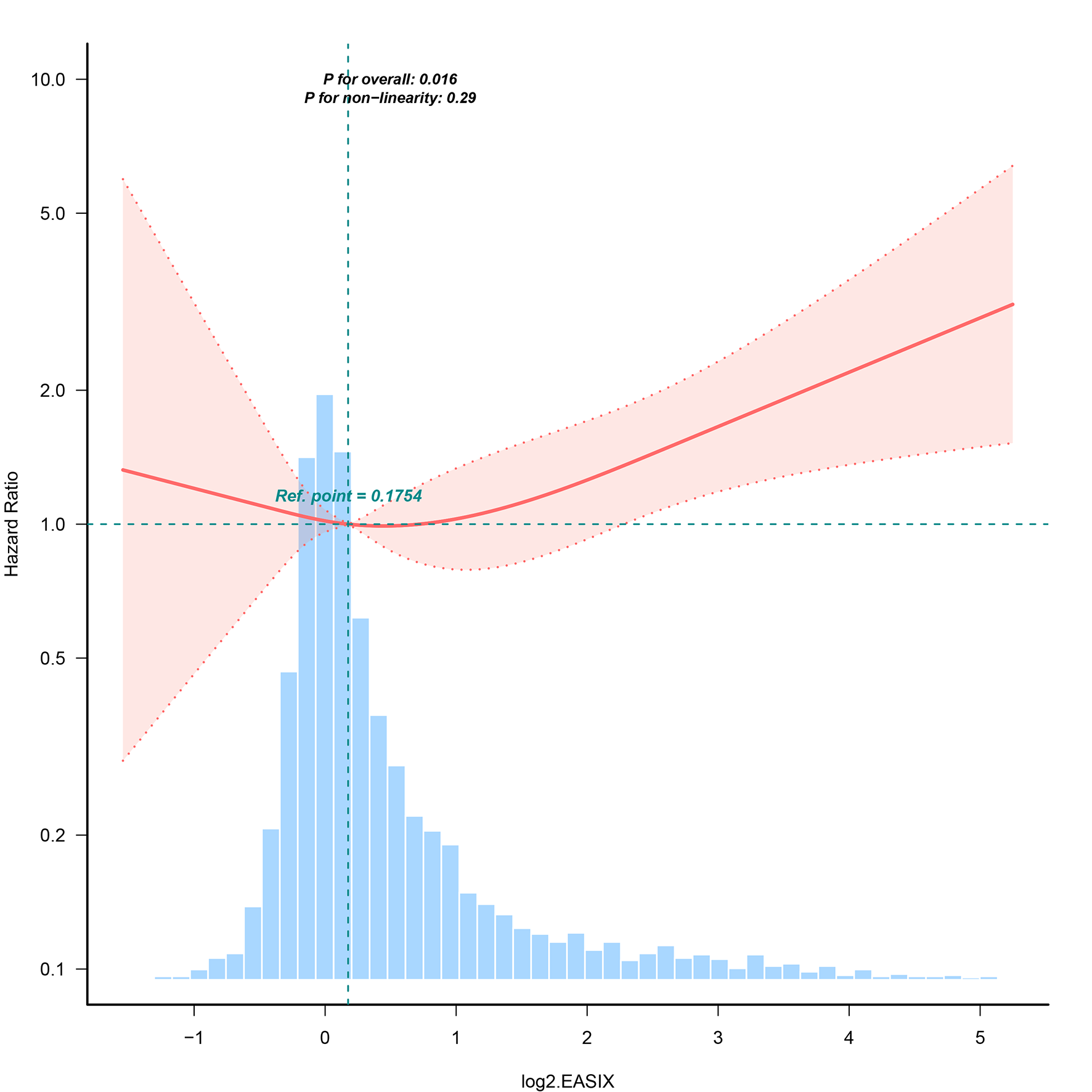

Supplement: Supplementary Figure 1 — Restricted cubic spline plot (Log2(EASIX) and 28-day mortality rate model). The variables that support the model include: Gender, age, BMI, heart rate, MBP, SPO2, charlson comorbidity index, apsiii, use hormone, use vasopressin, use ventlation, use rrt, wbc, neutrophils, lymphocytes, hemoglobin, BUN, PT, APTT, sodium, chloride, PCO2 and PO2. [file Image_1.tif]

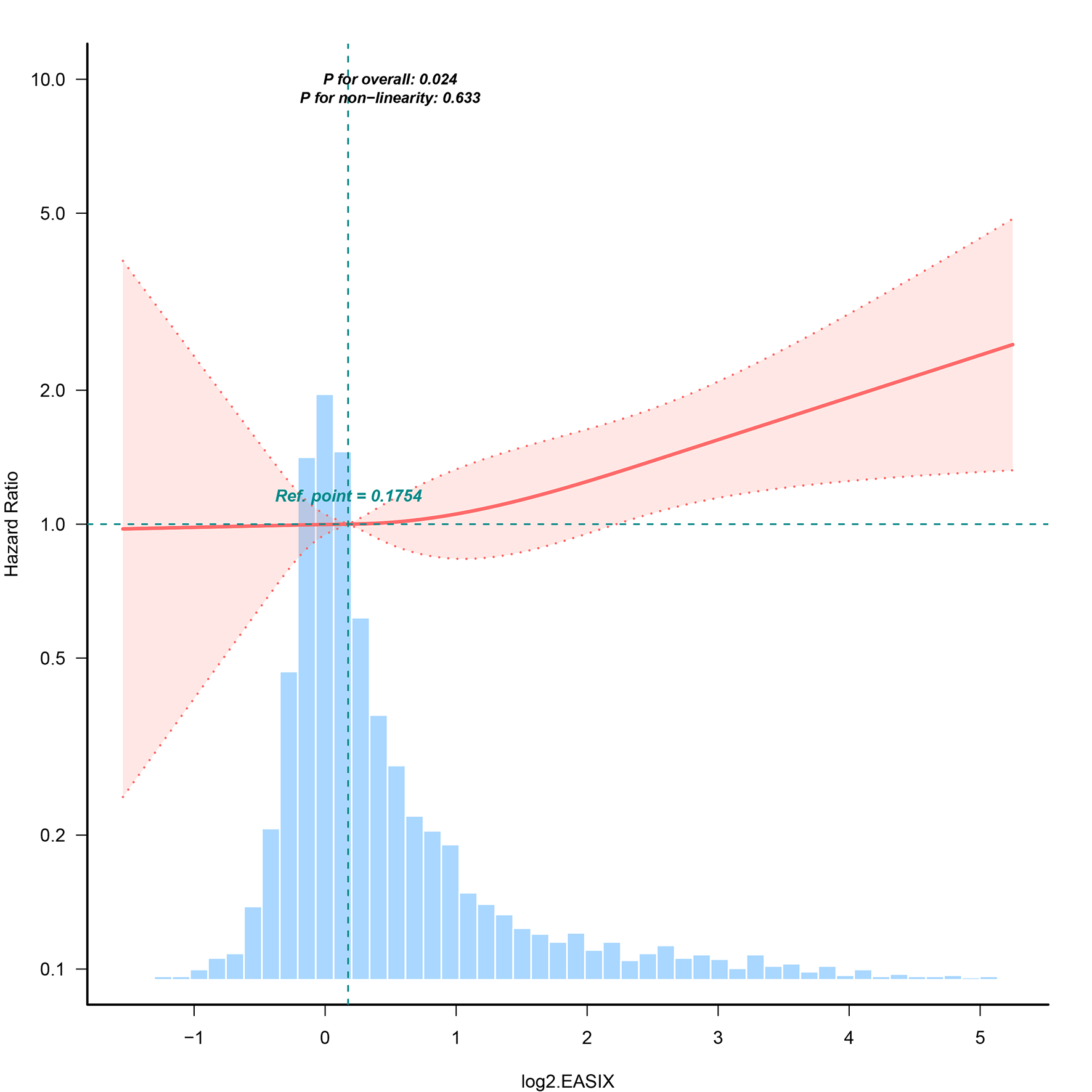

Supplement: Supplementary Figure 2 — Restricted cubic spline plot (Log2(EASIX) and 60-day mortality rate model). The variables that support the model include: gender, age, BMI, heart rate, MBP, SPO2, charlson comorbidity index, apsiii, use hormone, use vasopressin, use ventlation, use RRT, WBC, neutrophils, lymphocytes, hemoglobin, BUN, PT, APTT, sodium, chloride, PCO2 and PO2. [file Image_2.tif]

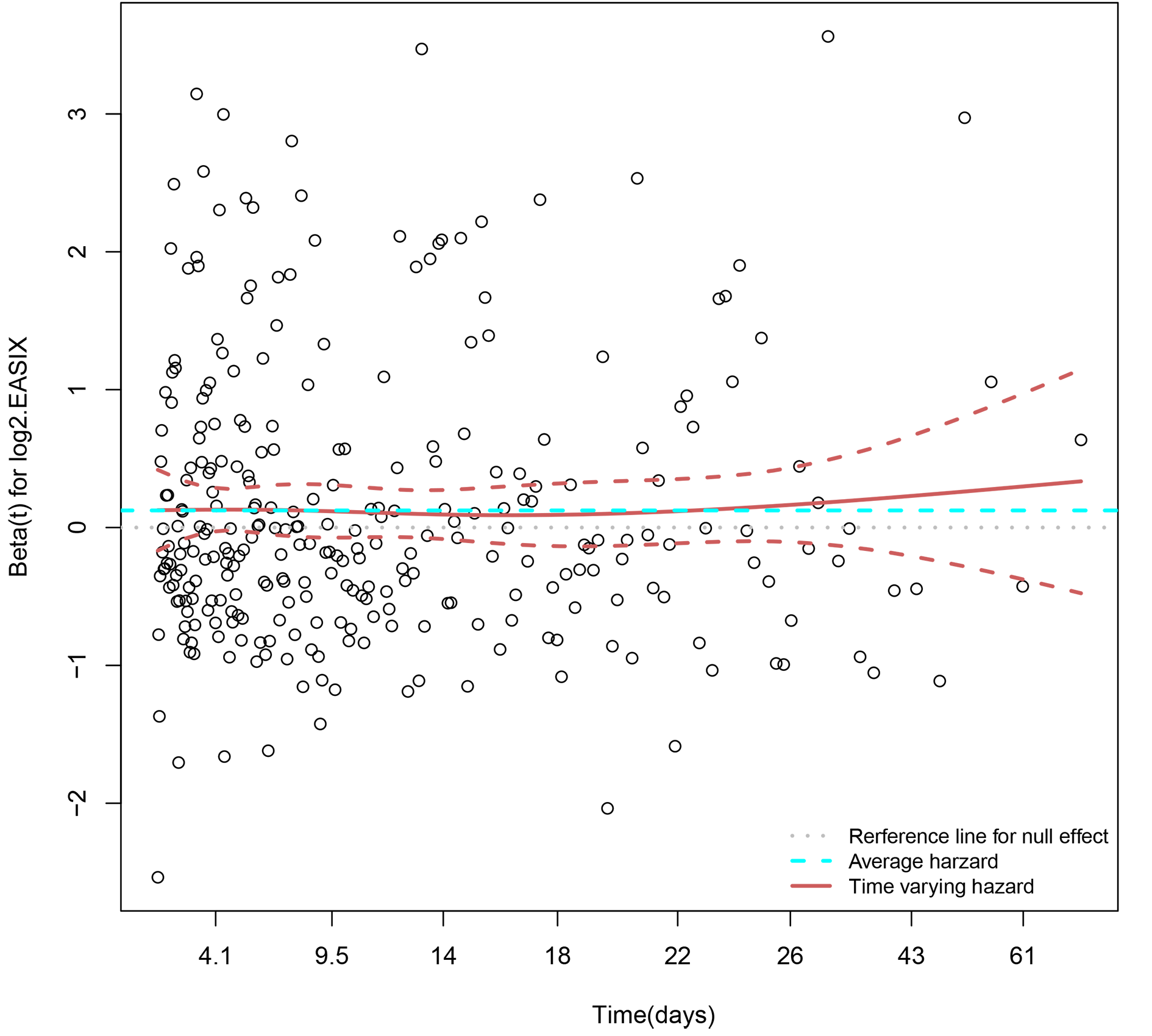

Supplement: Supplementary Figure 3 — Schoenfeld residual plot for Log2(EASIX) in relation to 28-day mortality. A 1-unit increment in log2(EASIX) corresponded to a time-independent 14% increase in 28-day mortality risk, supporting the proportionality of hazards. [file Image_3.tif]
